# Clinical application of artificial intelligence technology in epilepsy

**DOI:** 10.1186/s42494-026-00268-0

**Published:** 2026-08-10

**Authors:** Qi Ren, Yan Zhang, Xiaohong Deng, Meng Fu, Xia Li, Luyao Wang, Xianpeng Chen, Yu Yang, Jinfeng Zhang

**Affiliations:** 1 Affiliated Baotou Clinical College of Inner Mongolia Medical University, Baotou, Inner Mongolia 014040 People’s Republic of China; 2 Department of Neurology, Baotou Central Hospital, Baotou, Inner Mongolia 014040 People’s Republic of China

**Keywords:** Artificial intelligence, Epilepsy, Machine learning, Deep learning, Electroencephalography, Neuroimaging

## Abstract

Epilepsy is a prevalent neurological disorder, and its inherent complexity and significant interindividual variability pose substantial challenges for clinical diagnosis and treatment. Against this backdrop, the rapid development of artificial intelligence (AI) technology is driving a historic paradigm shift in the field of epilepsy diagnosis and management. This review analyses the application of AI in four core domains of epilepsy clinical practice: early diagnosis, accurate seizure prediction, individualized treatment, and long-term disease management. The primary objectives of this review are to delineate the current status of the clinical application of AI in epilepsy, clarify emerging future trends, and ultimately provide practical references and actionable insights for clinical practitioners.

## Background

Epilepsy, as a complex and common chronic neurological disorder characterized by transient, repetitive, and recurrent seizures, imposes substantial physical, psychological, social and economic burdens on patients themselves, their families and even society throughout the long-term treatment process [1–3]. The pathogenesis of epilepsy is associated with multiple neuropathological processes, such as ion channel dysfunction [4], inflammatory responses [5], physiological network dysfunction [6], and abnormal glial cell activity [7], although the exact mechanisms driving the disorder remain incompletely understood [8]. Data from the Global Burden of Disease Study [9] indicate that by 2021, approximately 52 million individuals globally had active epilepsy. Prevalence rates, however, differ markedly across nations, regions, and urban versus rural settings, indicating that epilepsy is among the top causes of mortality among individuals with neurological disorders [10].

With the continuous advancement of medical technology, novel diagnostic tools, including voxel-based morphological analysis techniques [11], stereo electroencephalography [12], and positron emission tomography-magnetic resonance imaging (PET-MRI) [13], have significantly enhanced the accuracy of epilepsy diagnosis. Concurrently, advancements in anti-seizure medications (ASMs) have improved the control of epileptic seizures in patients [14]. However, the efficacy of existing diagnostic and therapeutic approaches is limited by low diagnostic accuracy and insufficient individualized treatment strategies. Therefore, ensuring the accurate diagnosis and effective treatment of epilepsy has become a critical goal in current medical research.

**Fig. 1:**
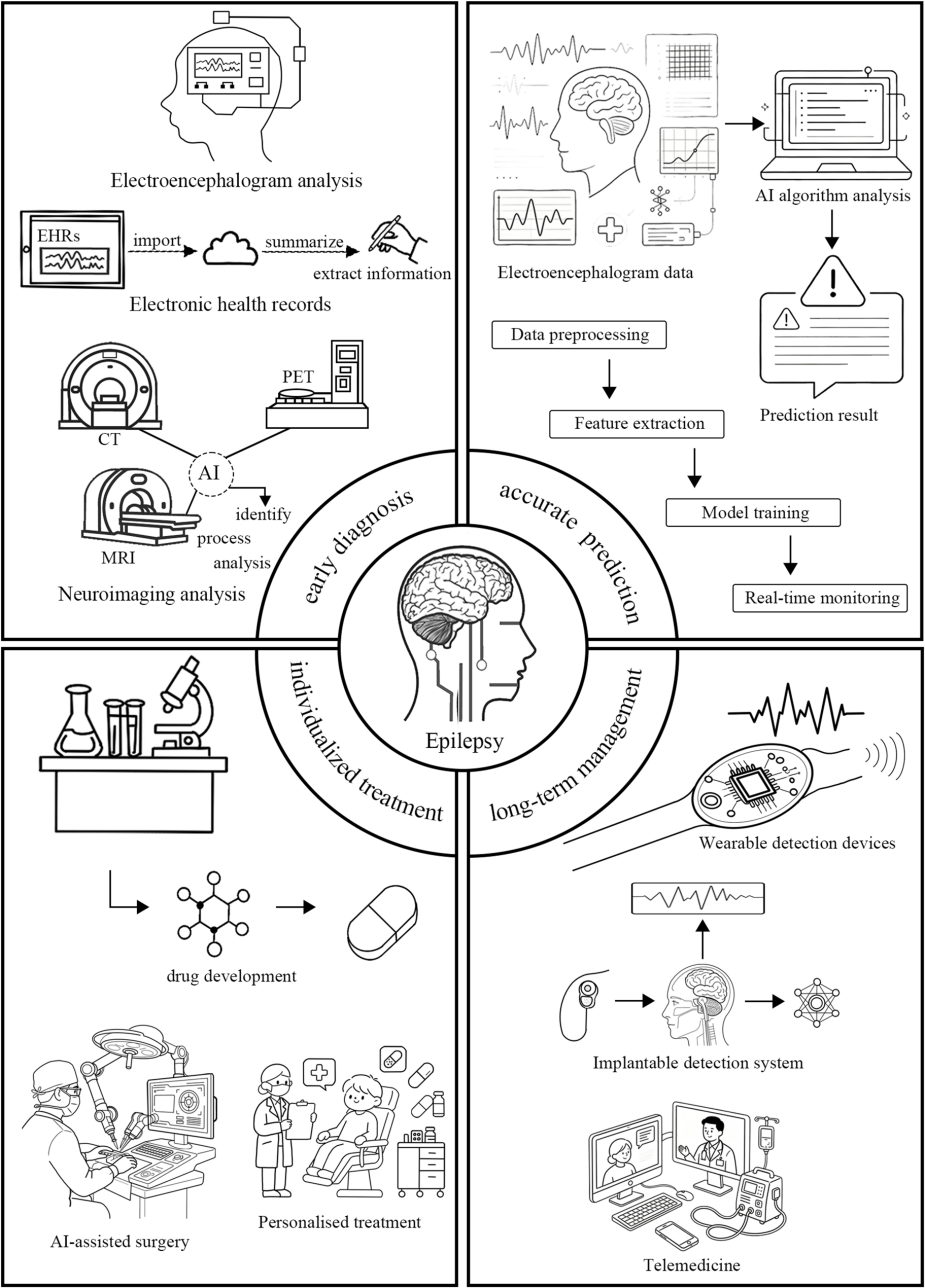
AI methods to the four clinical domains

AI can mimic human thought processes; enhance the precision and efficiency of medical practices [15]; and has been widely adopted across hospitals, clinical laboratories, and research institutions. In recent years, the rapid development of AI technology, particularly the application of machine learning (ML) and deep learning (DL), has profoundly affected diverse medical fields. Specifically, ML enables computers to learn from data, identify patterns via algorithms, and thereby generate predictions and decisions. DL, as a more advanced subset of ML, leverages multi-layer artificial neural networks to process and analyse large volumes of complex data [15–17]. Owing to their ability to be trained for pattern recognition and inference from data [18], AI algorithms have become vital tools for optimizing the clinical diagnosis and treatment of epilepsy. In summary, AI has broad application prospects in this domain. Therefore, this paper systematically presents the outcomes of AI technology in the clinical application of epilepsy, aiming to provide a reference for the diagnosis, prediction, treatment and long-term care of this disease (Fig. 1).

## Clinical applications of AI technology in epilepsy

### Applications in epilepsy diagnosis

#### Electroencephalogram analysis

Electroencephalography (EEG) is a powerful non-invasive neuromonitoring tool characterized by millisecond-level temporal resolution, cost effectiveness, and portability, making it well suited for clinical epilepsy monitoring [19]. It is among the most critical modalities for epilepsy diagnosis [20]. EEG plays a crucial role in supporting clinicians’ diagnostic workflows by identifying the epileptogenic onset zone, capturing real-time brain electrical activity, detecting structural abnormalities, and recognizing abnormal brain activity associated with epileptic seizures [21–23]. Video monitoring serves as a vital complement to EEG in epilepsy diagnosis, particularly for assessing the clinical manifestations of seizures. Video-EEG (an integrated modality combining video and EEG) provides more comprehensive multi-dimensional information, assisting clinicians in distinguishing epileptic seizures from non-epileptic events and determining the severity and classification of seizures during an episode.

In recent years, the application of AI technology in EEG analysis has increased substantially. By leveraging AI algorithms, real-time analysis of video data enables the identification and classification of epileptic seizures, thereby facilitating rapid determination of seizure type, duration, and severity. In particular, ML and DL, two core subsets of AI, significantly increase the accuracy and efficiency of EEG analysis and have now emerged as the optimal tools for processing and analysing EEG signals [24, 25]. Notably, DL algorithms, particularly convolutional neural networks (CNNs), a class of deep neural networks, excel at identifying spatial patterns in EEG signals and exhibit significant advantages in capturing features associated with epileptic activity [26, 27]. Moreover, a novel automated epilepsy detection method that integrates path signatures and a bidirectional long short-term memory (Bi-LSTM) neural network with an attention mechanism enables the extraction of long-term EEG-related information, thereby enhancing the accuracy of seizure detection [28].

These advanced algorithms hold substantial potential for assisting clinicians in epilepsy diagnosis by rapidly processing large volumes of EEG data. They not only improve the efficiency of epilepsy monitoring but also equip clinicians with more intuitive diagnostic evidence. Furthermore, these algorithms alleviate clinicians’ workload and shorten follow-up time, empowering them to formulate patient-specific treatment plans more efficiently [29].

#### Neuroimaging analysis

Neuroimaging plays a crucial role in epilepsy diagnosis, particularly through technologies such as structural magnetic resonance imaging (MRI) and positron emission tomography (PET), for identifying epilepsy-related lesions. However, traditional visual assessment often fails to detect subtle lesions [30]. AI algorithms address this by automating the analysis of MRI, PET, and functional imaging data, thereby enabling the automatic identification of lesions in patients with epilepsy and significantly improving lesion detection rates [31, 32]. A CNN-based model, for example, enables the automatic detection and classification of abnormalities in MRI data, facilitating the early detection and tracking of epilepsy [33]. Additionally, in one study, researchers developed an innovative ML algorithm—Subtype and Stage Inference that identifies different spatiotemporal trajectories of brain atrophy progression in patients with epilepsy, providing a novel rationale for the individualized prognosis and targeted treatment of epilepsy [34]. Furthermore, AI approaches integrating different imaging modalities, such as [^18^F] flurod eoxyglucose-PET and [^18^F] flumazenil-PET, exhibit significant predictive power for localizing epileptic foci [35].

#### Electronic health records analysis

The widespread adoption of electronic health records (EHRs) has provided a rich database for the diagnosis and management of epilepsy. Notably, unstructured data within EHRs represent a valuable source of medical information, facilitating centralized research on large-scale EHR datasets. Automated EHR phenotyping (AEP), a method that integrates features from unstructured records with structured data, enables the automatic identification of patients diagnosed with epilepsy. This ML-based approach can accurately identify epilepsy patients from clinical notes and anti-seizure medication (ASM) prescriptions, thereby facilitating large-scale epilepsy research leveraging EHRs. Additionally, AEP has potential for application in epilepsy phenotyping analysis within genetic research and precision medicine trials [36, 37]. Furthermore, natural language processing (NLP) technology can analyse unstructured data in medical records to identify symptoms related to epileptic seizures, treatment responses, and patients’ lifestyle factors. Combining NLP tools with traditional ML algorithms to extract information on patients’ epilepsy-related characteristics, treatment processes, and outcomes from EHRs offers robust support for clinical decision-making [38].

### Application in epilepsy prediction

AI has demonstrated significant advantages in epileptic seizure prediction. For instance, the Epi-AI model built on the XGBoost algorithm enables accurate semi-automated analysis of EEG data, resulting in high sensitivity and specificity in EEG assessments across multiple mouse epilepsy models. This capability enhances the reliability and reproducibility of epilepsy seizure analysis in rodent EEG datasets, thus providing a highly efficient tool for epilepsy research [39]. Yang Li proposed a multi-head attention multi-branch graph convolutional network for the automatic detection of paediatric epilepsy using EEG. This model integrates three types of graph convolutions—spatial distance, functional connectivity and adaptive graph—and combines them with separable multi-head attention to capture spatiotemporal dependencies, thereby providing an efficient and intelligent tool for the rapid clinical screening of paediatric epilepsy [40]. Moreover, another early prediction model for epileptic seizures based on long-term recurrent convolutional networks combines the spatial feature extraction capability of CNNs with the temporal modelling capability of long short-term memory networks. This integration allows accurate identification of the pre-seizure phase in EEG signals, ultimately reducing seizure-related risks and harms [41].

In 1972, Stevens et al. conducted long-term, multi-channel continuous EEG recordings on several patients with epilepsy and behavioural disorders and investigated the distribution of interictal spikes, seizure frequency and temporal patterns of seizures, thereby providing early neurophysiological evidence for seizure prediction. However, this method is limited by technical constraints, has a restricted scope of application, and struggles to accommodate the complexity of EEG signals and individual variations [42]. With the advancement of ML technologies, algorithms such as support vector machines and random forests have been introduced into epilepsy prediction. Researchers have established predictive models by manually extracting features such as spectral power and connectivity from EEG signals. Subsequently, DL techniques—such as CNNs and long short-term memory (LSTM) networks—have become mainstream, enabling the automatic extraction of spatiotemporal features from EEG signals without the need for manual feature engineering. In recent years, research has shifted towards multimodal data fusion; multimodal artificial intelligence integrates clinical, EEG and imaging data, playing a crucial role in epilepsy diagnosis, seizure detection, treatment efficacy prediction and prognosis assessment, and represents a key direction for precision medicine in epilepsy [43]. Concurrently, contrastive learning and ResNet have been employed to mitigate the issue of insufficient labelled data, significantly enhancing model performance in personalised prediction [44]. Closed-loop neural stimulation systems (such as responsive neurostimulation system), combined with AI algorithms, enable real-time monitoring and intervention of pre-seizure electrical activity, thereby significantly reducing seizure frequency [45].

Moreover, research on the prediction of epileptic seizures has benefited greatly from the creation and sharing of open benchmark datasets. These datasets provide a standardised platform for algorithm development, performance comparison and reproducibility studies. For example, Boston Children’s Hospital-MIT is currently the most widely used scalp EEG epilepsy dataset; it was collected by Boston Children’s Hospital and curated by Ali Shoeb’s team at MIT. The data, which originated from paediatric patients with refractory epilepsy who were monitored for up to several days following the discontinuation of ASMs, were used to record seizure characteristics and assess the feasibility of surgical intervention [46, 47]. The Temple University Hospital EEG Corpus, collected by Temple University Hospital in the United States, is also a significant open-access EEG dataset and is currently one of the largest publicly available clinical EEG datasets. It is particularly suitable for tasks such as epilepsy detection, EEG event classification, and automatic clinical EEG interpretation, providing rich data support for the development of efficient and accurate EEG analysis algorithms [48]. The Siena scalp EEG dataset is primarily used for brain-computer interface and neuroscience research. As it covers multiple subjects and multi-task scenarios, it has become a key resource for research on EEG signal analysis [49]. The EPILEPSIAE dataset is currently among the most comprehensive publicly available EEG datasets dedicated to epilepsy research. It is widely used in studies involving seizure detection, classification and prediction and serves as a crucial benchmark dataset for evaluating deep learning models and signal processing algorithms [50]. The Freiburg Intracranial Electroencephalography dataset originates from the Epilepsy Research Centre at the University of Freiburg in Germany, and the data that were collected via intracranial electrode implantation surgery were used to not only elucidate the mechanisms of epileptic seizures but also aid the development of intelligent diagnostic technologies [51]. Although the aforementioned datasets have driven algorithmic development, issues such as data heterogeneity, annotation uncertainty and performance variations across datasets remain major challenges facing the field [52].

Currently, the clinical deployment of epilepsy seizure prediction systems faces a fundamental methodological choice: whether to adopt a patient-specific approach or a cross-patient approach. These two paradigms differ significantly in terms of data requirements, algorithm design, performance, and clinical deployability. A patient-specific approach involves constructing a dedicated prediction model based on a single patient’s EEG or other physiological signal data; however, while offering high accuracy and personalisation, it is difficult to apply directly to other patients. Cross-patient approaches utilise data from multiple patients to train generalised models that learn the common characteristics of epileptic seizures. Through transfer learning or feature sharing techniques, these models can be adapted for different patients. While this reduces the costs of data collection and model training, enabling large-scale clinical deployment, prediction accuracy may be compromised for some patients because of individual variations. With the continuous advancement of AI, it will become possible to utilise cross-patient models as a foundation and enhance accuracy through personalised fine-tuning, thereby providing a more efficient and precise service for seizure prediction [53, 54].

### Application in epilepsy treatment

#### AI-empowering drug development

Drug development is a complex, interdisciplinary process that requires coordination across multiple fields, with its primary goal of alleviating symptoms and treating diseases by developing novel drugs. While drug development is fraught with challenges, successful drug launches offer new therapeutic hope to patients. Nevertheless, the development of novel drugs still faces multiple hurdles. Integrating AI technology into the drug development workflow can effectively reduce research and development (R&D) costs, shorten R&D cycles, and increase R&D success rates, laying a foundational framework for guiding the clinical application of drugs [55–57].

The application of AI technology in drug discovery has become increasingly widespread. Specifically, integrating ML, DL, network pharmacology, molecular docking, molecular dynamics modelling, and network toxicology, among other approaches, can effectively support drug discovery and clinical application [58, 59]. With respect to epilepsy treatment, approximately one-third of patients fail to achieve seizure control with current medications, imposing a severe financial burden on their families and making the development of novel drugs an urgent priority [60]. A key advantage of AI technology lies in its ability to process complex multidimensional datasets, thereby revealing the associations between drugs and biological targets and optimizing the drug design and screening process. To address the challenges in the development of ASMs, Xiaoying Lv and colleagues have established a virtual repositioning screening platform for ASMs that integrates deep learning with molecular docking technology, focusing on key targets encoded by genes involved in the pathogenesis of epilepsy, successfully identifying a large number of candidate drugs with potential antiepileptic activity. This provides a reliable computational biological basis for the repurposing of existing drugs and offers new strategies and candidate directions for the development of precision treatments for epilepsy [61]. AI also plays a significant role in other neurological disorders. For instance, one research team was the first to identify the biomarker enzyme PHGDH (which drives Alzheimer’s disease pathogenesis) and constructed its 3D protein structure using the AI tool AlphaFold3 [62]. Similarly, leveraging AlphaFold multimer prediction and virtual screening, another team identified FAM171A2 as a novel therapeutic target for Parkinson’s disease and potential small-molecule drugs, which are expected to delay Parkinson’s disease progression [63]. In the context of epilepsy, AI can also predict the therapeutic outcomes of ASMs early by analysing large volumes of biomedical data, which enables clinicians to adjust treatment strategies promptly in clinical practice, ultimately improving patient prognosis [15, 64].

#### AI-assisted surgery

Surgery is an effective intervention for drug-resistant epilepsy (DRE), particularly in patients with focal epilepsy, where accurate location of the epileptic focus is pivotal to surgical success. AI and DL algorithms can preoperatively analyse patients’ EEG and imaging data, such as MRI and functional MRI (fMRI), prior to surgery to identify the epileptic focus and assess its impact on adjacent brain tissue. This analysis not only provides a robust foundation for surgical planning but also assists surgeons in making precise real-time intraoperative decisions, thereby facilitating further localization of the ictal onset zone and ultimately improving surgical success rates. PET is primarily used in epilepsy research to detect hypometabolic regions associated with the epileptic focus; applying AI for in-depth PET image analysis enables more precise focus localization. fMRI is employed preoperatively to map language functional areas: AI algorithms analyse fMRI data from language-related tasks to accurately predict the language-dominant hemisphere. Integrating AI into epilepsy surgery workflows not only enhances surgical safety but also increases surgeons’ confidence in the procedure [15, 17, 65–68]. Furthermore, artificial intelligence can be used to assess surgical outcomes and prognosis by monitoring patients’ physiological data, thereby enabling timely adjustments to treatment plans and further improving patients’ quality of life. In the future, the application of AI technology in the surgical treatment of epilepsy will require further exploration and consideration.

#### Personalised treatment

Currently, the treatment of epilepsy includes pharmacological and non-pharmacological interventions, with the latter including resective surgery, neuromodulation, and dietary therapy. Therapeutic strategies should be tailored to distinct disease stages to ensure treatment precision. Owing to interindividual variability, it remains possible to predict which medication will yield optimal efficacy and thus serve as the first-line agent for a given patient. Additionally, no biomarkers enabling robust prediction of treatment response or risk of DRE exist in routine clinical practice [69, 70]. On the basis of data from 2,586 epilepsy patients at their initial consultation, Kyung-Il Park’s team used AI technology to predict treatment responses to different ASMs. The results showed that AI was moderately effective at predicting the efficacy of ASMs; monotherapy with sodium valproate and the combination of levetiracetam and carbamazepine yielded the best predicted outcomes, offering potential support for personalised drug selection in clinical practice [71]. AI provides a novel approach to personalized epilepsy treatment via analysis of patients’ complex clinical and genetic datasets. This strategy not only enhances predictive accuracy but also assists physicians in formulating more effective treatment plans that minimize side effects and improve patient prognosis [66], underscoring the need for extensive development and application of AI in personalized epilepsy treatment workflows.

### Application in epilepsy management

#### Wearable detection devices

With the rapid development of mobile devices and wearable sensors, wearable technology has emerged as a critical innovative tool for continuous seizure monitoring and epilepsy management [29, 72, 73]. These devices enable seizure detection by collecting the patient’s physiological data and leveraging AI to process these data and identify seizure precursors [74]. Notably, wearable devices improve seizure detection rates and facilitate differentiation between seizures and normal physical activity [75]. For instance, in one study, researchers presented a DL-based hardware architecture for real-time epileptic seizure detection and personalized adjustment, offering an efficient, low-power solution for clinical applications [76]. Additionally, wearable devices that integrate ML algorithms and AI enhances the accuracy of seizure prediction [74]. Specific DL models, for example, can analyse video data to identify subtle movements and facial expressions, thereby enabling accurate detection of epileptic seizures [77]. Nevertheless, while wearable devices show great potential in the management of epilepsy, they still require further refinement to improve their detection accuracy so that they can better serve clinical applications [72, 74].

#### Implantable detection system

Implantable detection systems represent a robust management strategy for patients with DRE. These systems involve the implantation of electrodes into the patient’s body to continuously record EEG signals, enabling precise identification of the onset and type of epileptic seizures. Notably, implantable devices, such as the NeuroPace Responsive Neurostimulation System (RNS), support real-time monitoring of brain activity and the delivery of automated electrical stimulation to interrupt ongoing seizures, thereby mitigating seizure burden and improving patients’ quality of life [78]. Furthermore, the latest implantable devices, such as bilateral subcutaneous EEG recording systems, have shown promising sensitivity and specificity for long-term seizure monitoring. By providing high-quality EEG data, these devices facilitate the development of more targeted treatment plans by physicians [79]. Additionally, recent research has proposed an epileptic seizure detection model based on tiny ML technology, which offers an efficient approach for implantable closed-loop neural stimulation systems. This model achieves real-time, high-precision seizure detection via analysis of iEEG signals, providing a valuable tool for personalized DRE management [80]. The primary advantage of implantable detection systems lies in the precision and real-time nature of their data, which allows physicians to better characterize patients’ seizure patterns and tailor treatment strategies accordingly.

#### Telemedicine

Telemedicine is an innovative approach that enables the exchange of medical information across geographic locations via electronic communication technologies, with the aim of improving patient health outcomes [81]. It allows clinicians to communicate in depth with patients, offering more flexible health care options and significantly enhancing health care accessibility [82]. Integrating AI into telemedicine systems—specifically by developing telemedicine platforms that incorporate open-source EHRs—can substantially reduce clinicians’ workload. This not only improves the accessibility of diagnostic services and the efficiency of epilepsy diagnosis but also enhances patient satisfaction, reduces seizure frequency, and ultimately achieves bidirectional synergistic development in clinical practice [83].

## Current state of AI technology in epilepsy

### Projects ready for clinical implementation

AI technology in the field of epilepsy has now progressed from basic research to routine clinical application; some technologies have undergone multi-centre validation, have received Food and Drug Administration (FDA) approval, and can be directly applied in routine clinical practice. The detection of non-convulsive seizures on EEG in intensive care units (ICU) is a clinical challenge; patients do not exhibit obvious convulsive symptoms, yet their EEGs show persistent abnormalities. If not treated promptly, this can lead to secondary brain damage. However, traditional routine EEG requires operation by specialist technicians and interpretation by remote experts, resulting in significant time delays. A study analysed Rapid-EEG data from 353 critically ill and emergency department patients across six hospitals in the United States and validated Claritγ, an FDA-approved machine learning-based real-time EEG monitoring AI system. It is capable of highly sensitive screening for non-convulsive status epilepticus, quantifying seizure burden, and rapid risk stratification, thereby reducing diagnostic delays and overtreatment. It can be directly applied in routine clinical practice and is a reliable tool for epilepsy monitoring in the ICU [84]. The interpretation of routine EEGs forms the basis of epilepsy diagnosis, but there are currently two major challenges: first, limited inter-expert agreement; and second, the uneven distribution of specialist resources. Jesper Tveit and colleagues developed and validated an AI model (SCORE-AI) capable of fully automated interpretation of routine EEG through a multi-centre study. With a specificity of 90% and an accuracy comparable to that of human experts, the model is ready for direct use in routine clinical practice. It has the potential to improve the efficiency of epilepsy diagnosis and treatment, reduce misdiagnoses, and enhance diagnostic consistency [85].

### AI technology for epilepsy that is not yet ready for widespread clinical use

Research into the application of AI in the diagnosis and treatment of epilepsy is growing exponentially, with a host of new technologies demonstrating promising potential in laboratory settings in terms of both technical feasibility and efficacy. However, a significant translation gap remains between laboratory and clinical settings. The vast majority of studies are currently at the stage of demonstrating technical feasibility and showing promising results in small, single-centre trials; however, they lack multi-centre external validation, suffer from limited generalisability, and lack standardised protocols, and are therefore not yet suitable for routine clinical implementation. ST-CANet is the first multimodal AI localisation system to integrate stereoelectroencephalography with structural connectivity. It has been validated in a single-centre clinical trial, demonstrating high localisation accuracy and strong reproducibility. It is suitable for routine preoperative assessment of drug-resistant temporal lobe epilepsy, thereby reducing reliance on clinical judgement and improving surgical precision. However, issues such as a small sample size and a lack of external validation present obstacles to its clinical translation [86]. Yipeng Zhang and colleagues developed and validated PyHFO, a lightweight, end-to-end deep learning software for high-frequency oscillation analysis. Designed for the automatic detection and classification of epileptogenic high-frequency oscillation (HFO) in intracranial EEG recordings, the software meets the requirements for routine clinical implementation and can be directly applied to preoperative assessment and intraoperative monitoring, providing a scalable AI solution for precision epilepsy surgery. Although technically feasible, PyHFO has not yet been validated in prospective, multicentre clinical trials to demonstrate its ability to improve clinical outcomes, and further work is needed to establish its clinical value [87]. These technologies represent future possibilities but should not be used to guide clinical decision-making.

### Projects in the theoretical exploration phase

Unlike mature technologies that have already received regulatory approval and laboratory techniques that have undergone single-centre validation, AI technologies at the theoretical exploration stage are at the very forefront of innovation and represent the future direction of precision treatment for epilepsy. Such technologies remain at the stage of animal experiments, in vitro models, proof-of-concept studies or computer simulations. Although they are innovative and feasible, they face technical bottlenecks, ethical risks and unproven safety and are still far from clinical translation. Yuyan Shen’s team utilized two open-source AI video analysis tools, DeepLabCut and B-SOiD, to automatically analyse videos of fluorane-induced seizures in 32 mice of different genetic backgrounds and in Angelman syndrome model mice. They extracted millisecond-scale motor micro-features, constructed a behavioural database and quantified behavioural complexity [88]. Jie Yang proposed a low-power, closed-loop neural modulation AI chip based on algorithm-circuit co-design, which enables highly sensitive, implantable real-time epilepsy detection and neural modulation with extremely low false-positive rates, thereby providing a mass-producible hardware solution for closed-loop epilepsy treatment. Although it meets the requirements for routine clinical implantation and precise intervention, it has not yet undergone long-term stability testing in chronic animal models. Furthermore, as the model was trained on limited data, its ability to adapt to different types of epilepsy and individual variations remains to be verified [89]. Zhichao Liang’s team proposed an online learning closed-loop neuromodulation framework based on the Koopman operator for real-time seizure prediction and precise electrical stimulation intervention. This addresses the shortcomings of traditional open-loop stimulation—namely, its limited efficacy and significant side effects—as well as the computational complexity and implantability challenges associated with existing AI models. This provides critical technical support for the transition of closed-loop neuromodulation from the laboratory to routine clinical practice. However, it has been validated using only computational models and retrospective EEG data, and no real-time closed-loop experiments have been conducted in animal models [90]. Mercy Edoho’s team developed a machine learning prediction system for a mouse model of amygdala-hypoxanthine-resistant epilepsy. By analysing short-term EEG data during seizure episodes, the system can predict the frequency of subsequent spontaneous seizures in mice in advance, thereby significantly reducing the need for manual screening, minimising animal wastage and accelerating drug development. This provides an efficient and scalable AI tool for the preclinical screening of ASMs. However, its accuracy is only 75%, posing a high risk of misclassification, and it requires validation using larger sample sizes and a wider range of model types [91]. Although the aforementioned cutting-edge AI technologies for epilepsy are not yet ready for clinical implementation, they have overcome the limitations of traditional treatments and point to more precise, personalised and non-invasive treatment approaches.

## Challenges in the application of AI technology in epilepsy

### Clinical translation and challenges

#### Learning challenges and algorithm validation issues in unbalanced EEG data

EEG data for epilepsy are often insufficient, collected from small-sample studies, and display imbalances across seizure types. These problems not only affect the training performance of deep learning models but also result in an incomplete verification system for the validity, generalisation and reliability of AI models related to epilepsy. Existing research confirms that while traditional machine learning classification algorithms demonstrate strong epilepsy detection capabilities on balanced experimental datasets, they are prone to issues such as overfitting to the majority class, high rates of missed seizures, and insufficient sensitivity when confronted with inherent class imbalance in real-world clinical EEG data. Consequently, relying solely on conventional classification models fails to meet clinical diagnostic needs, thereby limiting their practical clinical value. Comparative studies have also demonstrated significant differences in robustness among various traditional classification algorithms when applied to imbalanced EEG scenarios. Conventional models generally struggle to balance sensitivity and specificity, further highlighting the need for specialised imbalanced learning strategies tailored to epilepsy-related tasks [92–95].

#### Issues related to the regulation of medical devices

AI-based epilepsy diagnosis and monitoring systems are classified as artificial intelligence medical devices and must comply with relevant regulatory standards set by the FDA, Conformité Européenne (CE) and domestic medical device approval bodies. However, the industry currently faces challenges such as unclear market access pathways and difficulties in regulating algorithm updates. The US FDA has established a dedicated regulatory framework for AI/ML medical devices, which is applicable to intelligent diagnostic support systems such as automated EEG detection, seizure prediction and epileptogenic focus localisation. Among these, epilepsy AI monitoring and diagnostic systems are generally classified as low- to medium- or medium- to high-risk medical devices; those used for routine screening and real-time bedside alerts are mostly classified as medium-risk, whereas AI systems used for preoperative epileptogenic focus localisation and surgical planning guidance fall under the high-risk regulatory category. Under the Medical Devices Regulation, the European Union regulates AI medical products for epilepsy, with CE marking being a prerequisite for entry into the European clinical market. However, AI systems designed for long-term epilepsy monitoring and intraoperative lesion localisation currently lack clear guidelines for clinical efficacy evaluation, risk classification standards, and post-market surveillance mechanisms. Consequently, a significant number of research-grade algorithms struggle to achieve regulatory compliance and cannot be formally integrated into routine clinical practice.

#### Integrating real-world clinical challenges

First, the majority of medical professionals—including neurologists and EEG technicians—lack systematic training in the fundamentals of AI and specialised training in interpreting data results. This leads to the misuse of AI data, resulting in two extremes: either over-reliance or complete distrust, which hinders the routine application of artificial intelligence in the clinical management of epilepsy. Furthermore, the interpretation of epilepsy findings requires clinicians to conduct a comprehensive assessment by integrating EEG characteristics, patient history, and imaging data rather than relying solely on algorithmic outputs; this is a core issue that must be addressed when applying AI in clinical practice. Additionally, EEG signals are highly dependent on the subject’s physiological characteristics, hardware equipment, and recording environment. Differences in individual patient circumstances, EEG recording equipment and environmental conditions result in significant individual variability in the waveforms, rhythms and amplitudes of abnormal discharges, which is another key issue limiting clinical implementation. Finally, most real-time detection models have been validated only on public datasets, lacking generalisation testing across multiple centres, different devices and diverse populations; furthermore, there is low integration between the algorithms and existing hospital EEG workflows. Balancing real-time performance, accuracy, generalisability and clinical feasibility remains the core challenge in current research on real-time epilepsy detection. The diagnosis and treatment of epilepsy involves not only a purely technical process of identifying and intervening in pathological lesions but also a comprehensive assessment of the patient’s language function, emotional state, behavioural patterns, and other complex, context-dependent factors. Such a multifactorial, holistic analysis cannot rely solely on artificial intelligence [92, 96, 97].

### Ethical and safety risks

Most existing deep learning and ensemble learning models used for epilepsy detection and localisation are black-box models. These algorithms generally suffer from a significant lack of interpretability; they can only output diagnostic results without clearly presenting the basis for their decisions, key EEG features, or the logic behind channel contributions. Consequently, for reasons of medical safety and professional responsibility, clinicians find it difficult to rely on AI results as the primary basis for diagnosis and treatment and can use them only as an auxiliary reference. This not only makes it difficult to gain the confidence and acceptance of clinicians but also hinders the progress of medical device regulatory approval, ethical compliance and the standardisation of clinical trials for AI-assisted epilepsy diagnosis, thereby significantly limiting the application value of AI in high-value scenarios such as presurgical assessment and intensive care. These issues not only impede the clinical translation and application of AI models but also reduce clinicians’ willingness to use AI-assisted diagnosis. Furthermore, epilepsy research and clinical applications rely on vast amounts of patient EEG signals, medical records, imaging data and sensitive personal health information. Throughout the entire process—from data collection, transmission and annotation to model training and cross-institutional sharing—there are privacy risks associated with data leaks, unauthorised reuse and excessive data collection. Furthermore, the absence of unified standards for data de-identification, authorised use and storage security makes it difficult to balance the need for open access to research data with the protection of patient privacy, presenting an ethical challenge that requires resolution. Consequently, the optimal integration of AI technology into clinical epilepsy treatment remains an area that requires further exploration [97, 98].

## Conclusions

With the continuous maturation of AI technology and its deeper integration into clinical practice, AI holds vast prospects in epilepsy diagnosis and treatment, especially in advancing precision medicine for epilepsy. For clinicians, mastering AI technology and its capabilities and leveraging them in clinical practice enables effective improvements in both patient outcomes and quality of life. Looking ahead, through the systematic, comprehensive integration of AI into clinical workflows, clinicians are expected to deliver more precise, individualised health care services to epilepsy patients. This will ultimately facilitate the achievement of precision medicine for epilepsy while providing patients with enhanced diagnostic and therapeutic experiences and outcomes.

## Data Availability

No datasets were generated or analysed during the current study.

## Abbreviations

ASMs: Anti-seizure medications
AI: Artificial intelligence
ML: Machine learning
DL: Deep learning
EEG: Electroencephalography
CNNs: Convolutional neural networks
LSTM: Long short-term memory
MRI: Magnetic resonance imaging
PET: Positron emission tomography
EHRs: Electronic health records
AEP: Automated EHRs phenotyping
NLP: Natural language processing
fMRI: Functional MRI
R&D: Research and development
DRE: Drug-resistant epilepsy
FDA: Food and drug administration
ICU: Intensive care unit
CE: Conformité européenne
